# Identification of microplastics using 4‐dimethylamino‐4′‐nitrostilbene solvatochromic fluorescence

**DOI:** 10.1002/jemt.23841

**Published:** 2021-05-28

**Authors:** Giuseppe Sancataldo, Vittorio Ferrara, Francesco Paolo Bonomo, Delia Francesca Chillura Martino, Mariano Licciardi, Bruno Giuseppe Pignataro, Valeria Vetri

**Affiliations:** ^1^ Dipartimento di Fisica e Chimica – Emilio Segrè Università degli Studi di Palermo Viale delle Scienze, 18 Palermo Italy; ^2^ National Interuniversity Consortium of Materials Science and Technology (INSTM) UdR of Palermo Florence Italy; ^3^ Dipartimento di Scienze e Tecnologie Biologiche Chimiche e Farmaceutiche Università di Palermo Viale delle Scienze, 17 Palermo Italy; ^4^ ATEN Center Università degli Studi di Palermo Viale delle Scienze, 18 Palermo Italy

**Keywords:** DANS, environmental pollution, microplastics, phasor analysis, spectral analysis

## Abstract

In this work, we introduce the use of 4‐dimethylamino‐4′‐nitrostilbene (DANS) fluorescent dye for applications in the detection and analysis of microplastics, an impendent source of pollution made of synthetic organic polymers with a size varying from less than 5 mm to nanometer scale. The use of this dye revealed itself as a versatile, fast and sensitive tool for readily discriminate microplastics in water environment. The experimental evidences herein presented demonstrate that DANS efficiently absorbs into a variety of polymers constituting microplastics, and its solvatochromic properties lead to a positive shift of the fluorescence emission spectrum according to the polarity of the polymers. Therefore, under UV illumination, microplastics glow a specific emission spectrum from blue to red that allows for a straightforward polymer identification. In addition, we show that DANS staining gives access to different detection and analysis strategies based on fluorescence microscopy, from simple epifluorescence fragments visualization, to confocal microscopy and phasor approach for plastic components quantification.

## Graphical abstract

- 4‐Dimethylamino‐4′‐nitrostilbene (DANS) fluorescent dye allows microplastics staining.
- Under UV, DANS‐stained microplastics glow a solvatochromic emission.
- Fluorescence emission signal allows for a straightforward plastic identification.

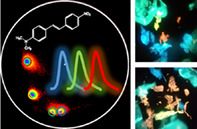

## 1 INTRODUCTION

In the last century, plastics production has progressively increased, since these materials have succeeded as constituents of most of the everyday life objects. The plastics use supports breakthrough sustainable technologies in high relevance areas, such as transports, smart and efficient building, food conservation and medical sectors, but their large‐scale production has been leading to continuous accumulation of plastics dismissed waste (Barnes, Galgani, Thompson, & Barlaz, 2009; Ivar Do Sul & Costa, 2014). In this scenario, the plastic materials overproduction has determined an alarming presence of ubiquitous plastic pollution in aquatic environments, glaciers, soils, and even in the atmosphere, becoming a worldwide problem (Andrady, 2011; Chen, Feng, & Wang, 2020; Wright, Thompson, & Galloway, 2013). In addition to the accumulation of large visible debris, the presence of small plastic particles of size in the microscale and nanoscale used in industrial processes, commercial products, or originating from the degradation of daily use objects have been reported as a real danger for both the ecosystem and human health (Anbumani & Kakkar, 2018; Smith, Love, Rochman, & Neff, 2018). These small plastics, whose size ranges from millimeter (below 5 mm in size) to nanometer scale, are named Microplastics (MPs), mainly consisting of synthetic organic polymers. Due to chemical inertness of these polymers, a long environmental persistence of the plastics residues is observed, so that the small size MPs enter the food chain as internalized by living organisms, which are a primary source of food for humans (Desforges, Galbraith, & Ross, 2015; Silva‐Cavalcanti, Silva, de França, de Araújo, & Gusmão, 2017). In fact, the presence of MPs has been detected in mineral water, sea salt, sugar, honey, and several kinds of marine organisms, such as fish, jellyfish, and algae (Rainieri & Barranco, 2019). Although MPs contaminations have also been found in terrestrial ecosystems, the marine organisms are currently the most investigates species to elucidate the MPs effects on living organisms' biological functions, since they are reported to be strongly exposed to plastic debris, and could represent the main route for plastic particles introduction in the human diet (Wang, Gao, Jin, Li, & Na, 2019). In addition of their direct effects, MPs are supposed to be potential vehicles of toxic chemicals and pathogenic microorganisms as well, that can interact and bind on the MPs polymeric surface, then be delivered into the superior organisms, resulting in chemical and biological detrimental effects (De‐la‐Torre, 2020). Therefore, MPs are of special concern as they have been shown to induce different adverse effects, which still need to be further unveiled (Hwang et al., 2020; Ryan, 2015; Sharma & Chatterjee, 2017).

In the last few years, large efforts of the scientific community have been focused in the characterization of their main sources and pathways, with the aim to reduce their impact on the ecosystems, then on the human health (Rochman, 2018). Identification of MPs and their origin may help planning littering prevention solution of most found items into the environment (Sun, Dai, Wang, van Loosdrecht, & Ni, 2019).

In this context, a growing literature exists aimed to develop analytical methods to identify and characterize MPs (Hidalgo‐Ruz, Gutow, Thompson, & Thiel, 2012; Prata, da Costa, Duarte, & Rocha‐Santos, 2019). Indeed, there is an urgent need to increase our specific knowledge about these heterogeneous and complex mixtures of polymers and additives. The understanding of MPs impact can be reached through the knowledge of their accumulation in specific areas, along with the characterization of the plastics micro‐/nanoparticles in terms of size, shape, chemical composition, fragmentation, and degradation pathways.

Although several analytical strategies have been applied in MPs investigation (Mariano, Tacconi, Fidaleo, Rossi, & Dini, 2021), no method has been adopted as a standard analytical approach up to date (Prata, Alves, da Costa, Duarte, & Rocha‐Santos, 2020). MPs identification usually consists in a preliminary visual examination of the sample often supported by the bright‐field optical microscopy (Lusher, Welden, Sobral, & Cole, 2017). This first step is largely dependent on operator and is quite time consuming. The identification procedure generally goes on with the analysis of their chemical composition by means of mass spectrometry, Fourier transform infrared (FTIR) and Raman spectroscopies, often aided by thermoanalytical methods (Käppler et al., 2016). In particular, FTIR and Raman spectroscopies implemented in imaging mode have revealed high potentiality allowing for noninvasive identification and characterization of MPs by mapping chemical properties of the sample with microscale spatial resolution (Harrison, Ojeda, & Romero‐González, 2012; Tagg, Sapp, Harrison, & Ojeda, 2015). Although these methods provide a large amount of information, including possible presence of pollutants and modifications due to environmental conditions, they may be time consuming especially for the analysis a relevant number of particles (Anger et al., 2018). Furthermore, due to spatial resolution limits, they may be less effective for the identification of MPs with size in the submicron scale leading to inaccurate observations such, for example, the underestimation of MPs contamination levels in the samples. Analytical low time‐consuming methods with minimal specimen preparation are largely desired which may allow to map and quantitatively characterize this heterogeneous system, simultaneously giving the possibility of monitoring biochemical processes or MPs interactions with living systems. An ideal method would enable to characterize and distinguish MPs, to readily quantify their morphological properties, to identify the polymeric constituents, and to monitor degradation effects. Ideally, in situ 4D (x,y,z,t) measurements in different matrices should be accessible of samples coming from seawater, soils, and biological tissues.

In this context, fluorescence microscopy has emerged as an ideal tool for these applications providing fast high‐resolution analysis of MPs through the use of fluorescent dyes, small organic molecules whose spectral properties critically change depending on the interaction with the environment (Cole, 2016). When interacting with MPs, some fluorescent dyes allow the plastic fragments visualization with high signal to noise ratio. In specific cases, such an interaction can be also used to gain relevant information concerning changes in the dye absorption and fluorescence spectra, quantum yield, and fluorescence lifetime, that can be related to the MPs composition (Prata, Reis, et al., 2019; Sancataldo, Avellone, & Vetri, 2020). In this framework, a large number of studies have focused on the use of the Nile Red (9‐diethylamino‐5H‐benzo[a]phenoxazine‐5‐one) fluorescence, as a cheap and easy tool for the identification of MPs in environmental samples, also accessing automated analysis (Erni‐Cassola, Gibson, Thompson, & Christie‐Oleza, 2017; Hengstmann & Fischer, 2019; Prata, Reis, et al., 2019; Tamminga, 2017). Nile red stains the MPs resulting in fluorescent plastic fragments, whose emission varies from yellow to red according to the polarity of the constituent polymers (Maes, Jessop, Wellner, Haupt, & Mayes, 2017; Shim, Song, Hong, & Jang, 2016). The classification of MPs according to their polarity represents a useful property to be assessed as related to important features of the materials.

Moreover, analytical fluorescence‐based experiments at increasing level of complexity can be also designed to investigate MPs physicochemical modifications by mimicking different environmental degradation processes, for example, sunlight exposure, O_2_/water‐mediated oxidation, thermally induced modifications. Thus, it is noteworthy that the use of fluorescent dyes may have feasible applications in the MPs analysis, as the particles can be easily detected, counted, and classified by means of a wide‐field fluorescence microscope, a common instrumentation widely present in many laboratories. This approach can be carried out for MPs detection in water and sediments samples, as well as in biological matrices by means of confocal microscopy. In particular, concerning the biological samples, the fluorescence microscopy has stood out and affirmed as one of the principal technique for noninvasive analyses of tissues and cells, with consolidated protocols that can be directly translate for the MPs detection in living organisms, toward a more comprehensive knowledge of the MPs biochemical fate and their effects on human health.

Since fluorescence microscopy and related quantitative techniques in MPs research are evident promising analytical tools, the identification of other MPs interacting fluorescent dyes and their spectroscopic properties is a key point.

In this work, we show how the dye 4‐dimethylamino‐4′‐nitrostilbene (DANS) allows discriminating different MPs, with high selectivity, according to their polarity. DANS is a small fluorescent molecule whose emission spectrum is known to dramatically redshift as the polarity of its microenvironment increases (Lakowicz, 2006). Its positive solvatochromic behavior is known since the 1970 and it is attributed to charge transfer mechanisms which stabilize the molecule in the excited state (Reichardt, 1994). Physicochemical properties leading to changes in DANS fluorescence are then analogous to the ones regulating Nile Red. However, the spectral shift of DANS emission spans over a larger wavelength range, respect to Nile Red, providing larger sensitivity (Lakowicz, 2006). Proof of concept of the analytical applicability of DANS fluorescence to the identification of MPs from common use plastic items is given in this work, using five different model plastics: polypropylene (PP), low‐ and high‐density polyethylene (LDPE and HDPE, respectively) polystyrene (PS) and polyethylene terephtalate (PET). These plastics are among the most commonly produced (~80% of worldwide production) and their presence in form of MPs is highly abundant both in marine and in freshwater ecosystems (Shahul Hamid et al., 2018).

Our results clearly indicate that DANS solvatochromic behavior confers to plastics different fluorescence emissions, spanning from blue to red, over the entire visible range, once the sample is illuminated with UV‐light. We show that a qualitative screening of MPs is made easier as the MPs assume distinguishable colors, which underlies significant differences both in terms of shape and position of the DANS fluorescence emission band, making the use of the dye well suited for the application of spectral phasor analysis. The coupling of DANS staining with spectral phasor analysis provides a simple graphical method for the rapid identification and counting of MPs in the samples. We present different analytical strategies based on DANS fluorescence microscopy, from simple epifluorescence fragments visualization, to confocal microscopy and phasor approach for plastic components.

## 2 MATERIALS AND METHODS

### 2.1 Sample preparation

MP fragments were prepared scraping fragments from blocks of commercial virgin plastic using a metal file (USAG990 B PA/200). Microsized samples were collected and stored in separated tubes. Consumer items of PP (recycling code 05), HDPE (recycling code 02), LDPE (recycling code 04), PS (recycling code 06), and PET (recycling code 01) were used. Plastics block were identified based on their recycling symbols (Worrell & Reuter, 2014).

A 500 μM stock solution was obtained dissolving DANS (Sigma Aldrich, product number 39255) in ethanol. For MPs staining, 50 μl of DANS stock solution were added to a 25 mg/ml suspension of MPs in deionized water, to obtain a final DANS concentration of 25 μM.

Samples were incubated for 1 h at 60°C under vigorous agitation.

### 2.2 Attenuated total reflection–FTIR

An FTIR spectrometer (Bruker ATR FTIR, model ALPHA) in attenuated total reflection (ATR) mode, equipped with a diamond measurement interface and controlled by OPUS software, was used to collect IR spectra. Spectra have been acquired in the range 4,000–400 cm^−1^ with a resolution of 2 cm^−1^. Each measurement is the result of the average of 64 scans. The ATR diamond crystal was cleaned with 70% ethanol/water and a background measurement was performed between each sample. Sample was compressed against the diamond to ensure good contact between sample and ATR crystal.

### 2.3 Steady‐state fluorescence

Steady‐state fluorescence measurements were carried out on a JASCO FP‐8500 spectrofluorimeter at room temperature. All samples dispersed in water were placed in a 1 cm path plastic disposable cuvette (BRAND) and spectra were measured under excitation at *λ*_exc_ 405 nm using 0.5 nm wavelength step intervals, with excitation bandwidth of 5 nm and emission bandwidth of 10 nm using scan‐speed of 100 nm/min and integration time of 1 s. To avoid MPs sedimentation, a crosshead magnetic stirring bar (BRAND) was used for dispersing the microsized sample in the water solution (400 rpm). RGB pictures of stained sample, under UV excitation (LED 395 nm, Eletorot), were simply recorded by a phone camera (Huawei P20). The autofluorescence of each sample was checked and it is null or neglectable in all presented experimental conditions.

### 2.4 Fluorescence confocal microscopy

Confocal images were acquired with an Olympus FluoView1200 confocal laser scanning microscope (Olympus, Japan) using a 10 × 0.3 NA objective. Aliquots of DANS‐stained MPs dispersed in water were deposited on a cover glass (BRAND #1) using a disposable pipette. Measurement was acquired using laser excitation at 405 nm. Emitted fluorescence was acquired in photon‐counting mode. Spectral detection has been performed using a bandwidth of 5 nm and a step size of 3 nm in the range 420–740 nm. The scan area was 256 × 256 pixels and the scan speed was 12 μs per pixel.

### 2.5 Phasor analysis

The spectral phasor is a global algorithm for spectral imaging allowing the analysis of the heterogeneous emission of fluorescence molecule in images. It enables to map and characterize spectral features of multiple dyes or to detect spectral shape variations. The detailed description of the method and its applications is reported in Fereidouni, Bader, and Gerritsen (2012); Fereidouni, Bader, Colonna, and Gerritsen (2014).

**FIGURE 1:**
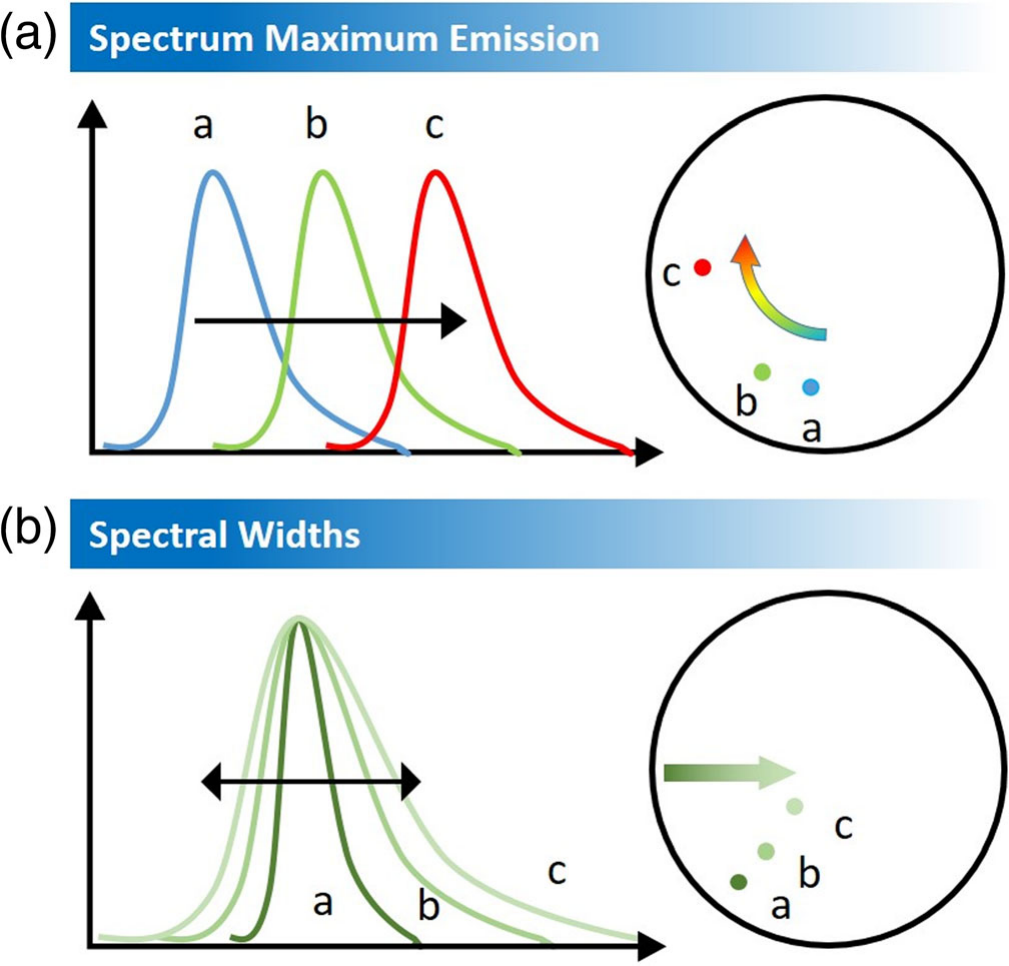
The spectral phasor plot for fluorescence emission spectra with different maximum emission wavelengths and spectral widths. (a) The phasor moves clockwise within the semicircle as the emission maximum moves from blue to red. (b) As the width of the spectrum increases the phasor moves toward the center

Briefly, in this work, reported spectral phasors are obtained from the first harmonic of the Fourier transformation of the spectrum measured at each point (pixel) of a fluorescence microscopy image. The real and the imaginary components of the transform, calculated according to the following definitions, are the X and Y component of the phasor which is plotted in a polar plot:

$$X=\frac{\sum_{\lambda }I\left(\lambda \right)∙\cos\left(2\mathrm{\pi n\lambda}/L\right)}{\sum_{\lambda }I\left(\lambda \right)};\;Y=\frac{\sum_{\lambda }I\left(\lambda \right)∙\sin\left(2\mathrm{\pi n\lambda}/L\right)}{\sum_{\lambda }I\left(\lambda \right)}$$

where *I* (*λ*) is the measured intensity at each step of the spectrum, *L* is the amplitude of the spectral range and *n* = 1. In the presented work, step is 2.5 nm and *L* is 320 nm. Each pixel in the spectral image contains the emission spectrum at that pixel. Therefore, an emission curve is associated with every pixel. From the definition, X and Y coordinated may assume values in the range between −1 and 1 thus the phasor lies within a circle. The angular position of the point on the phasor plot reflects the spectral position of the mass center of the emission spectrum while the distance from the center is inversely related to the spectral bandwidth. Importantly, phasors follow the vector algebra making simple the estimation of the contributions different species or the track of spectral modifications. A simplified vademecum for the interpretation of the spectral phasor analysis is reported in Figure 1.

Here, the phasors of fluorescence emission spectra with different maximum emission wavelength (Figure 1a) and spectral width (Figure 1b) are shown. A redshift of the emission spectrum is represented by a clockwise rotation in Fourier space represented by the circle. By increasing the width of the spectrum, the phasor moves toward the center of the circle. Here, we use the spectral phasor approach to monitor DANS interactions with MPs and to quantify the spectral position of the fluorescence emission due to specific polymers polarity.

### 2.6 Image analysis

Images were analyzed by ImageJ software (version 1.47v) using Java 1.6.0 (Schneider, Rasband, & Eliceiri, 2012). Phasor analysis of spectral images was performed using “Spectral Phasor plugin” of ImageJ developed by Fereidouni et al. (2012, 2014).

## 3 RESULTS

MPs used in this study were obtained by uniformly filing commercial plastic objects identified by the standard numbered symbols according the International Resin Identification Coding Systems (Worrell & Reuter, 2014). These plastics were used as models to proof that the analytical use of DANS fluorescence properties allows fast and reliable identification via the fluorescence color they assume, after staining, due to the spectral properties of this dye.

**FIGURE 2:**
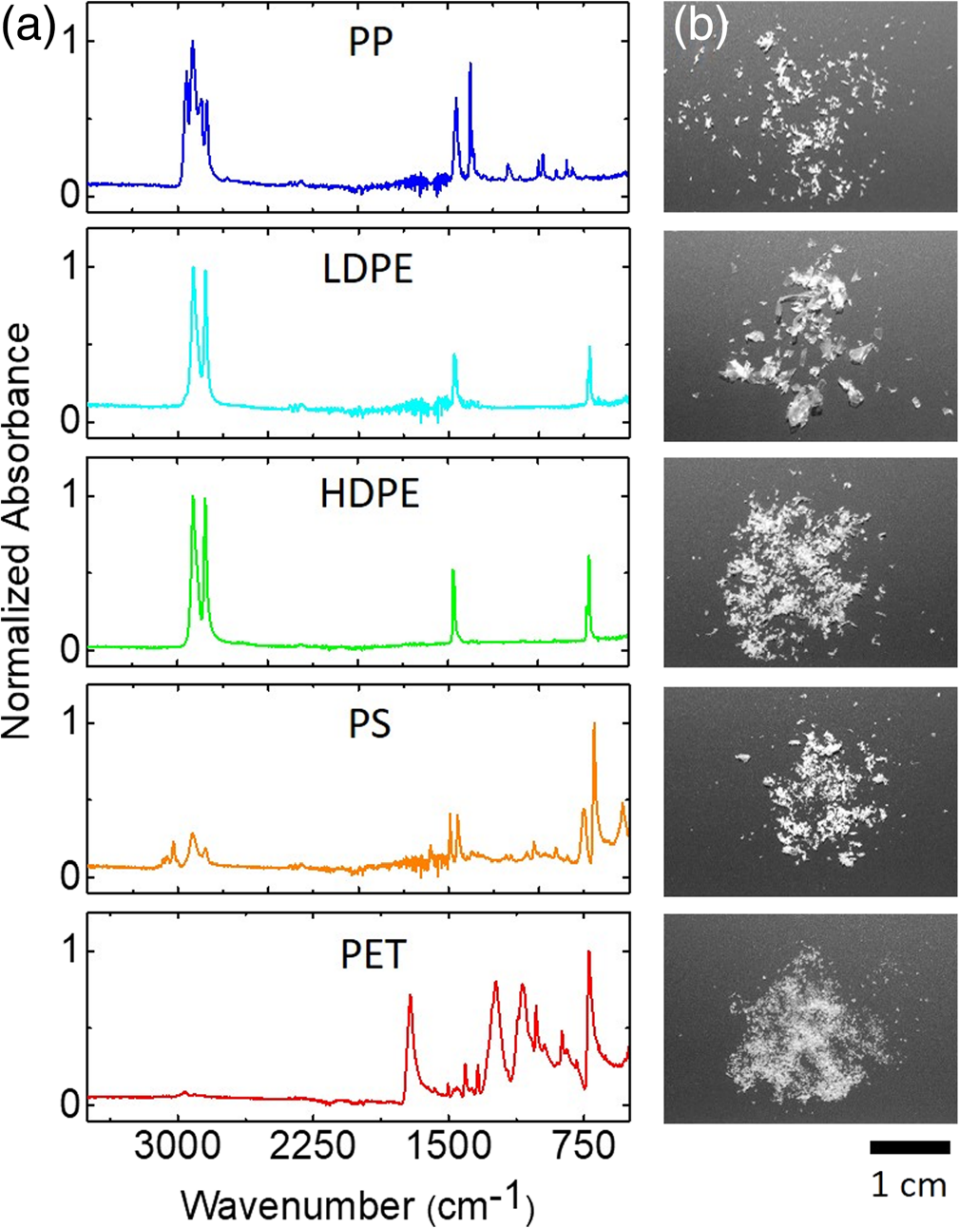
(a) Normalized attenuated total reflection (ATR) spectra of polypropylene (PP), low‐density polyethylene (LDPE), high‐density polyethylene (HDPE), polystyrene (PS), and polyethylene terephtalate (PET) microsized plastics obtained by fragmentation from plastic consumer goods with known resin codes. (b) Sample appearance to visual inspection

In Figure 2a, we report representative ATR–FTIR spectra of PP, LDPE, HDPE, PS, and PET microsized plastics obtained by the fragmentation process. These particles appear to the naked eye as homogeneous ground powders as shown in Figure 2b. Infrared spectroscopy is, nowadays, recognized as an efficient method for identifying plastic polymers as their absorption peaks are well known and easily distinguishable. Infrared analysis provides fingerprint spectra of the specific polymer of plastic material, as well as it may reveal the possible presence of other components (contaminants, additives, or polymer blends) (Jung et al., 2018). This method is currently applied in the identification of MPs in different environments (Tagg et al., 2015). Normalized ATR spectra acquired for each sample are reported in absorbance mode in the range 3,500–400 cm^−1^ and present expected absorption peaks previously reported in literature (Jung et al., 2018). Characteristic absorption bands (cm^−1^) used to identify each polymer is reported in Table 1. These bulk measurements confirm MPs composition and as no spare peaks are detected; it is possible to exclude significant contamination of the plastics within the experimental error.

**TABLE 1:** Characteristic IR absorption peaks (cm^−1^) used to identify each polymer

PP	2,915; 1,455; 1,377; 997; 982
LDPE	2,915; 2,845; 1,462; 717
HDPE	2,915; 2,845; 1,462; 717
PS	3,024; 2,847; 1,492; 1,451; 694
PET	1,713; 1,241; 1,094; 720

Abbreviations: HDPE, high‐density polyethylene; LDPE, low‐density polyethylene; PET, polyethylene terephtalate; PP, polypropylene; PS, polystyrene.

The same samples where stained with DANS fluorescent dye as described in the experimental section and dispersed in water. As reported in the introduction DANS is an archetype of solvatochromic molecule whose spectrum shifts to longer wavelengths and critically changes its shape as the polarity of its environment increases because of the stabilization of the dipolar excited state (Lakowicz, 2006). This feature already revealed its potential in the identification of different types of plastics using Nile Red fluorescence (Maes et al., 2017; Prata et al., 2020; Prata, da Costa, et al., 2019; Prata, Reis, et al., 2019; Sancataldo, Avellone, & Vetri, 2020; Tamminga, 2017) a widely used fluorescent dye for MPs fast identification using fluorescence microscopy. Nile Red spectral emission shifts toward higher wavelengths spanning from yellow for more hydrophobic plastics like PP and PE to red when in interaction with more polar plastics like nylon or PET (Maes et al., 2017; Sancataldo, Avellone, & Vetri, 2020).

**FIGURE 3:**
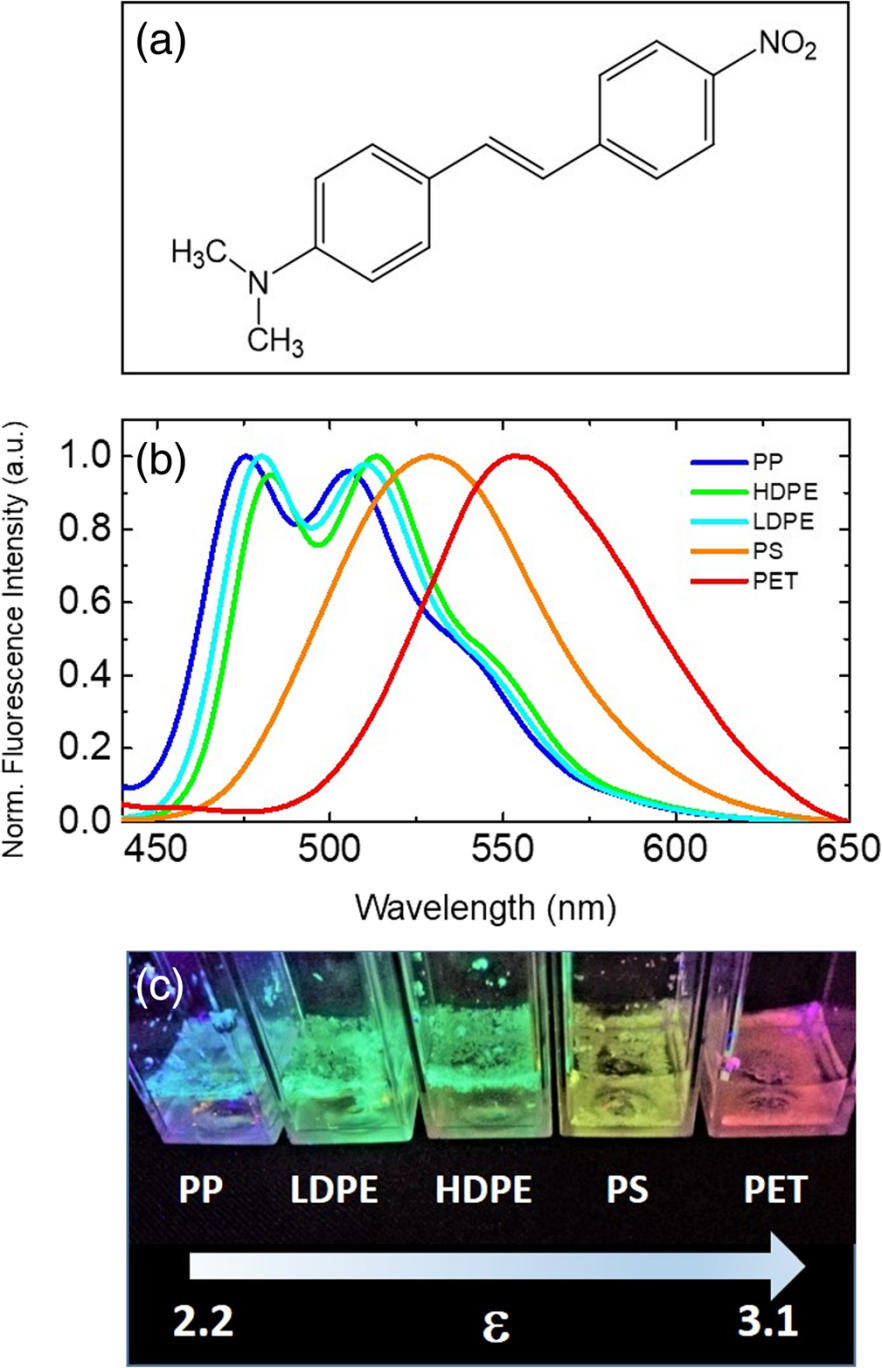
(a) Chemical structure of 4‐dimethylamino‐4′‐nitrostilbene (DANS). (b) Normalized steady‐state fluorescence emission spectra of polypropylene (PP) (blue), low‐density polyethylene (LDPE) (cyan), high‐density polyethylene (HDPE) (green), polystyrene (PS) (yellow), and polyethylene terephtalate (PET) (red) stained with DANS and dispersed in water. (c) Representative picture of DANS‐stained PP, LDPE, HDPE, PS, and PET microplastics in water illuminated using UV led. The arrow indicates increasing polarity of microplastics. Plastics are ordered according to the dialectic constant for the corresponding material: PP (2.2.–2.5), HDPE (2.3–2‐4), LDPE (2.2–2.35), PS (2.4–3.1) and PET (above 3) as a reference (Polymer Properties Database, 2020)

In Figure 3a, the molecular structure of DANS is reported. In Figure 3b, the steady‐state fluorescence emission spectra of DANS‐stained samples of PP (blue), LDPE (Cyan), HDPE (green), PS (yellow), and PET (red) acquired in bulk using *λ*_exc_ = 405 nm, normalized to their maximum, are reported. Representative pictures of the samples under UV illumination are also shown in Figure 3c. Fluorescence properties of DANS are due to intramolecular charge transfer between the dimethyl amino group (donor) and the nitro and carbonyl groups (acceptors) and the critical differences in the measured spectra reveal the expected differences in MPs polarity. As can be seen, the fluorescence emission peak red shifts according to the polarity of the MPs samples from the nonpolar sample made of PP particles to the one with higher polarity PET. The spectra of the analyzed samples span in the visible range from blue to red and critical differences between most of the plastic samples are evident. Polyolefins (PP and PE polymers) are non/less polar MPs and present two main peaks between 450 and 520 nm while polar MPs samples present a wide emission peak with a single maximum above 520 nm. Minor but significant differences in the presented experimental conditions are observed between HDPE and LDPE MPs samples. This observation is not surprising since both samples contain polyethylene MPs which only differ in the degree of branching, possibly the polymer molecular weight and its distribution. Nevertheless, the observation of minor differences between PE polymers confirms the sensitivity of the dye to MPs composition. The differences observed in fluorescence spectra are also visible to the naked eyes, DANS stained PP, PE, PS, and PET illuminated using a commercial portable UV lamp are clearly distinguishable by their color, from blue to red. The measured and detectable fluorescence colors of the MPs sample form a “MPs rainbow,” this feature possibly representing a major advantage with respect to analogous already confirmed sensing methods for the analysis of MPs using Nile Red whose spectrum modification are limited in the yellow‐red range. The wider spectral range, indeed, certainly has the potential to increase the ease of plastic discrimination providing a larger comparison scale.

**FIGURE 4:**
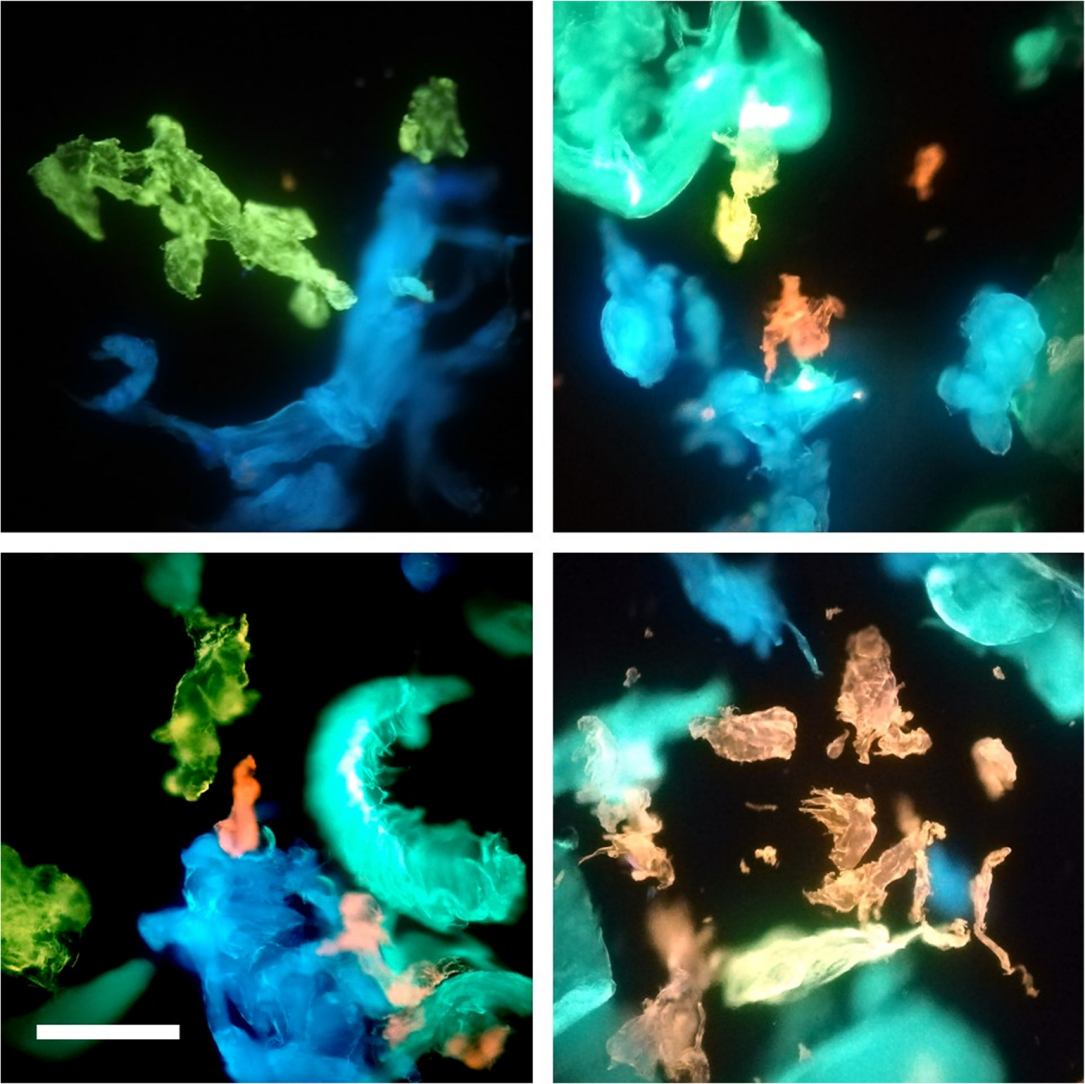
Representative epifluorescence microscopy measurements on water dispersions of polypropylene (PP), low‐density polyethylene (LDPE), high‐density polyethylene (HDPE), polystyrene (PS) and polyethylene terephtalate (PET) microplastic samples. 4‐Dimethylamino‐4′‐nitrostilbene (DANS)‐stained PP, LDPE, HDPE, PS, and PET microplastics in water illuminated using UV led. Scale bar is 300 μm

To further show the high potential of the application of this dye for applications in plastic discrimination, we report in Figure 4 representative epifluorescence microscopy measurements on water dispersions of PP, LDPE, HDPE, PS, and PET MP samples. Importantly, even a nontrained eye may readily select different MPs with a qualitative differentiation by their color. As can be seen, under UV illumination stained MPs reveal their “true” color: PP MPs are characterized by blue emission, LDPE and HDPE by green emission whit a slight deviation toward yellow of the latter, PS emission is clearly yellow while the high polarity of PET confers to MPs a brilliant red fluorescence.

This characteristic may be really important for the setup of a fast qualitative and portable selection method based on a simple portable fluorescence microscope.

In addition, it is important to note that although the large differences revealed in the visible color of the stained MPs, the spectroscopic features of the observed fluorescence certainly may give the chance to be used in more complex systems also allowing quantification of the number, size, and relative amount of different components in real samples. To do this it is possible to exploit (among the others) the possibility nowadays accessible to many commercial fluorescence confocal microscopes of easily acquiring fluorescence emission spectra of analyzed samples simultaneously with imaging. A standard measurement using a confocal microscope consists in the map of fluorescence intensity at every pixel of the image I(x,y) which, as it is well known, can be extended to 3D giving I(x,y,z). Spectral imaging extends the capabilities of fluorescence imaging studies combining the selectivity and sensitivity of spectroscopy with visual information and spatial resolution providing the map of the intensity measured at each spatial position as a function of the measured wavelength with defined wavelength steps thus giving I(x,y,z,λ). It should be noted that even if the combination of the fluorescence spectroscopy and microscopy is technically not trivial; nowadays, these analytical measurements are implemented in user‐friendly systems and readily accessible to many scientists. In addition, the analysis of the resulting data can be simplified using the spectral phasor approach (Fereidouni et al., 2012, 2014; Golfetto, Hinde, & Gratton, 2014). As described in the experimental section this method allows to transform the fluorescence emission spectra measured at each pixel of the image to a point in a polar plot (phasor) whose coordinates are the real and imaginary parts the of the first harmonic of the Fourier transform of the fluorescence emission spectra. This method was originally developed for spectral demixing of multiple fluorescence signals (Fereidouni et al., 2012, 2014; Golfetto et al., 2014; Malacrida, Gratton, & Jameson, 2015) but revealed also an high potential as it allows the graphical interpretation of change in spectral shape and position of environment sensitive fluorescent dyes in a simple and straightforward manner. Briefly, spectral images are translated in a polar plot in which the position of the phasor represents main feature of the detected fluorescence spectra: the average position of the fluorescence spectrum is associated with the angular position of the point on the phasor plot while its axial position is inversely related to its width. Pixels with similar spectra are grouped in cloud of close points which can be selected using colored cursors to the original fluorescence image, thus providing segmentation based on pixels with similar spectral properties.

**FIGURE 5:**
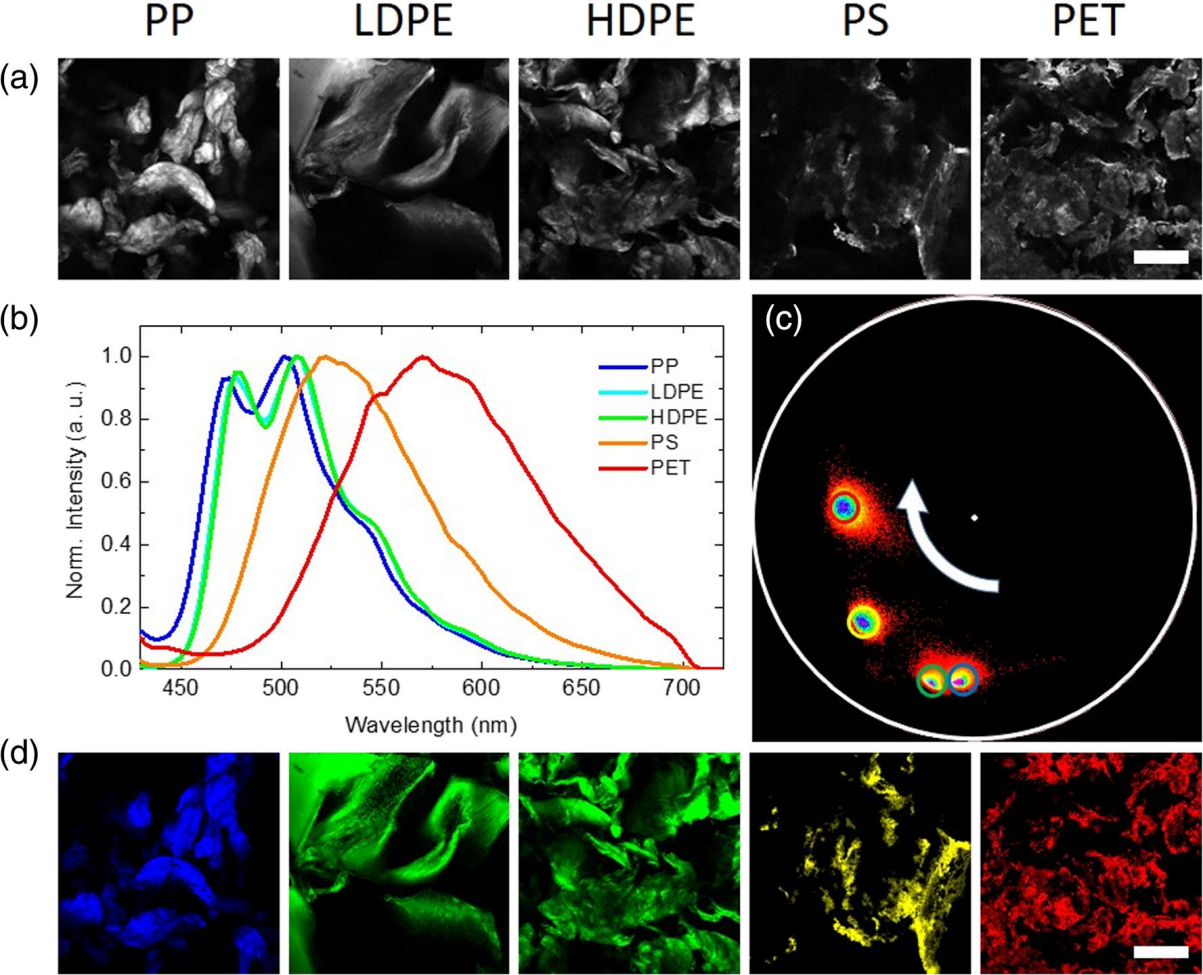
Spectral phasor analysis. (a) Representative 256 × 256 intensity‐based fluorescence confocal images of polypropylene (PP), low‐density polyethylene (LDPE), high‐density polyethylene (HDPE), polystyrene (PS), and polyethylene terephtalate (PET) obtained under excitation at *λ*_exc_ = 405 nm in the range 420–740 nm. Scale bar is 300 μm. (b) Fluorescence spectra obtained for the previous images PP (blue), LDPE (cyan), HDPE (green), PS (yellow), and PET (red) under the same laser excitation and in the same range. (c) Spectral phasor plot where the position of each phasor represents main characteristics of the emission spectral measured at the corresponding pixel; the red shift of 4‐dimethylamino‐4′‐nitrostilbene (DANS) spectrum is highlighted by the change in the phasor position from the bottom right along a clockwise direction. Pixels where fluorescence presents analogous spectral features are grouped in clouds, selected with colored circles: PP (blue), LDPE/HDPE (green), PS (yellow), and PET (red) and mapped back in the selection map (d). Scale bar is 300 μm

In Figure 5, we report representative spectral phasor analysis of spectral imaging experiment on the PP, HDPE, LDPE, PS, and PET MPs stained with DANS. In Figure 5a, 256 × 256‐pixels intensity‐based confocal fluorescence microscopy measurements of these sample acquired in the range 420–740 nm (under excitation at *λ*_exc_ = 405 nm) are reported. The field of view of the image is 1,272 μm × 1,272 μm. The acquired spectra measured in the same range for the presented images are represented in Figure 5b. The spectral phasor plot obtained from data analysis is reported in Figure 5c together with a color‐coded map where pixels selected with colored cursors are identified in the corresponding position.

As can be seen in Figure 5a, the plastic fragments are easily detectable and present uneven size and shape and quite heterogeneous intensity in each image. In these, measurement is not possible to recognize different MPs by visual inspection. As expected, measured fluorescence spectra, in this experimental setup, resemble the ones acquired in bulk using a standard spectrofluorimeter, of course due to technical aspects, these measurements are characterized by lower accuracy in the definition of the spectral shape and it is not possible distinguishing low and high density PE MPs samples whose signal are superimposed thus not allowing their separation. However, it is important to focus on the real strength point of these measurements which consists in the fact that the contribution to the measured fluorescence arises from few tens of MPs and that the contribution of each pixels in the reported images can be distinguished and traced. In line with measured spectra in bulk acquired spectra of DANS‐stained sample, even if spanned in a wide range and with clearly defined maxima position are largely overlapped so that classical spectral unmixing methods may require large analysis efforts. For this reason to separate pixels characterized by different fluorescence we report in Figure 5c the phasor analysis of the reported measurements. As can be seen four different clouds of point are clearly distinguishable. The different clouds were selected using a colored circle according to the previously defined color code of PP (blue), LDPE and HDPE (green), PS (yellow) and PET (red). Interestingly, the different pixel clouds are well separated with no or minimal overlap with the obvious exception of the HDPE/LDPE. In the phasor diagram, as the DANS spectrum position moves toward higher wavelengths due to the increasing polarity of the selected plastics, the position of the spectral phasor point moves in a clockwise direction. Moreover, the wider is the spectrum the lower is its distance from the phasor diagram center, this is more evident for the PET spectrum. These features allow clear and unambiguous discrimination of the spectral origin with pixels resolution. Pixels selected in the phasor plot with the colored cursors are mapped back in the images in Figure 5d so that the different MPs are distinguished by their true color.

**FIGURE 6:**
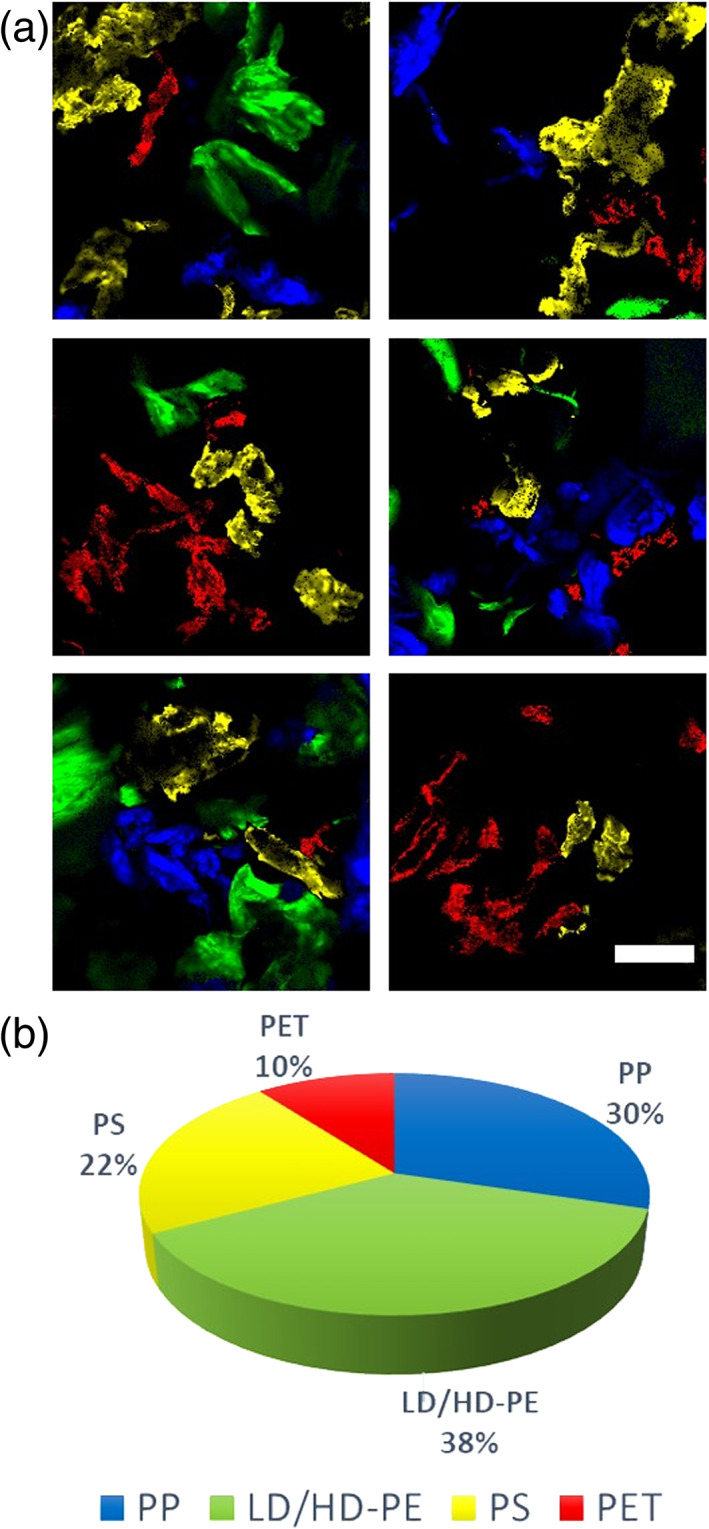
Detection, identification, and quantification of different microplastics: (a) Representative images of a mixture of microplastics simultaneously detected and colored according to the color code defined in the phasor plot reported in Figure 5c: polypropylene (PP) (blue), low‐density polyethylene (LDPE)/high‐density polyethylene (HDPE) (green), polystyrene (PS) (yellow), and polyethylene terephtalate (PET) (red). Scale bar is 300 μm. (b) Relative fraction of PET plastics detected in the images with respect to the total amount of detected plastics in the sample

To proof the possibility of distinguishing and quantifying different MPs in the same sample we report in Figure 6a the results of analogous measurements and analysis on a sample obtained by mixed MPs. A mosaic collection of images is shown to enhance the field of view.

Using the very same selections as the ones reported in the phasor plot in Figure 5c different MPs are quickly identified in the same images. Structures colored in blue are identified as PP, structures colored in green are polyethylene MPs, PS and PET are colored in yellow and red, respectively. A quantitative analysis of the relative fraction of the different plastics with respect to the total amount of MPs detected by the phasor analysis is reported in Figure 6b.

## 4 DISCUSSION

Results presented in this work clearly define DANS dye as a good candidate for the simple analysis of small MPs, it was shown that fluorescence signal of this dye may change along the whole visible spectrum from blue to red according to MPs polarity. Data reveal the potential of enhancing fast characterization of MPs via visual inspection adding a further parameter that can be performed as preliminary step before or in parallel to chemical characterization as micro‐FTIR or micro‐Raman methods. The high sensitivity and selectivity of fluorescence phenomena is well known and fluorescence has few competitors in sensing applications, by measuring fluorescence, indeed, it is possible to reveal fluorescent molecules down to nanomolar concentrations and to obtain suitable information on their environment and their modifications. Fluorescence measurements, and fluorescence microscopies in particular, is an already worldwide applied methods for MPs discrimination and quantification (Maes et al., 2017; Prata, da Costa, et al., 2019; Prata, Reis, et al., 2019; Sancataldo, Avellone, & Vetri, 2020; Tamminga, 2017). In particular, the use of the Nile Red fluorescent dye is rapidly wide spreading; since it first's applications, it was immediately clear that a wide size‐range of MPs could be quickly identified. The sample including MPs of different origins and, for example, transparent ones that usually constitute a challenge for the identification based on visual inspection using other optical microscopies (Erni‐Cassola et al., 2017).

We here present results using a less known small fluorescent molecule with solvatochromic properties. DANS is a fluorescent dye whose fluorescence emission spectrum modifications are due to analogous mechanisms to the one reported for Nile Red. For both dyes, the identification is mainly based on the spectral red shift which occurs with increasing polarity of the dye environment allowing classification of MPs in large chemical groups based on fluorescent shift (Reichardt, 1994). With respect to Nile Red, DANS may provide a wider comparison scale due to the larger accessible stokes shift range.

In this work, presented measurements are obtained with a really simple staining protocol which requires one‐hour incubation of the sample in water at 60°C with a similar protocol to the standard ones used for Nile red. DANS stained MPs emit visible light with high efficiency and their color allows the qualitative and quantitative identification of PP, HDPE, LDPE, PE, and PET samples without the use any filter even by naked eye. Of course to distinguish different individual MPs in different size ranges requires the use of lenses (and possibly filters) in fluorescence microscopy setup as shown in Figures 4 and 5. Nowadays, suitable microscopes for these applications can be obtained adding suitable lens to smartphone cameras, these low‐cost, portable experimental setups may open the way to new methods of fast prescreening of samples even in specific sites out of the lab. By increasing the complexity of the experimental hardware, it is possible to gain growing amount of information and reducing the size of detectable MPs and to obtain quantitation of MPs in terms of size, number, and chemical composition.

## 5 CONCLUSIONS

In conclusion, we herein presented the possibility to use the fluorescent dye DANS for the sampling, the detection and the analysis of MPs. Different levels of fluorescence‐based screening have been shown, from the simple identification of the MPs by the naked eye to the more complex phasor approach, which allows for a more refined analysis able to better distinguish the fluorescent signals even in case of similar polarity. In particular, the near‐UV illumination of DANS‐stained MPs results in glowing plastics fragments that clearly show different colors spanning from blue to red as a consequence of the different DANS emission spectra, which change according to MPs polarity. Differences in spectral features can be further exploited using fluorescence microscopy, which may allow to readily distinguish, count, and measure MPs by detecting the fluorescence signals from the sample in different spectral ranges. In addition, we also proposed the analysis of the spectral images obtained by fluorescence confocal microscopy to improve the performance of our approach on the analytical point of view. Spectral phasor was elected as preferential analysis method as it allows separating pixels characterized by similar fluorescence spectra, with the aim to overcome limitations due to partial spectra overlap. Moreover, it provides a simple and straightforward strategy to monitor and quantify both shape modifications and spectral shifts occurring in different spatial region of the sample.

As a future perspective, DANS staining, combined with the application of the spectral phasor method, can be considered as an emerging analytical strategy for chemical detection of the polymeric components constituting MPs detected in food and environmental samples. As well as this method can be used as a way to monitor environmentally relevant physicochemical processes occurring in MPs, such as polymer chemical degradation, or chemical and biological surface contamination, that are expected to induce spectral changes. Additional future developments could advance this approach with other advanced imaging systems (De Luca et al., 2020; Sancataldo, Anselmo, & Vetri, 2020; Sancataldo, Avellone, & Vetri, 2020) to allow for the identification, localization analysis, and tracking of individual types of MPs (and nanoplastics) from the (sub)cellular level (Duocastella et al., 2017; Sancataldo et al., 2017) to whole organs (D'Amora et al., 2016; D'Amora, Rodio, Sancataldo, Diaspro, & Intartaglia, 2019; Lavagnino et al., 2016) such as the brain (Sancataldo, Silvestri, Allegra Mascaro, Sacconi, & Pavone, 2019) and neural system (Gavryusev et al., 2019; Ricci et al., 2020; Sancataldo, Gavryusev, et al., 2019). Indeed, depending on the experimental setup, especially on the numerical aperture of lenses, plastics fragments of size ranging from millimeter size down to light diffraction limit (≈200 nm) can be analyzed (Diaspro, 2016; Pawley, 2002). As a consequence, the presented approach can address the increasing demand of a reliable analytical strategy for nanoplastics analysis as well, a cutting‐edge aspect in the context of environment and food plastic contaminants detection, whose interest is currently rising up due to the nanoplastics impact on the human health.

## CONFLICTS OF INTEREST

The authors declare no conflicts of interest.

## AUTHOR CONTRIBUTIONS

**Giuseppe Sancataldo** and **Valeria Vetri**: Developed the idea behind the project and designed the experiments and were responsible for the work planning, and management. **Giuseppe Sancataldo** and **Vittorio Ferrara**: Performed the experiments. **Giuseppe Sancataldo**: Analyzed the data. **Francesco Paolo Bonomo**, **Delia Francesca Chillura Martino**, **Mariano Licciardi**, and **Bruno Giuseppe Pignataro**: Provided critical instrumental and analytical expertise. **Valeria Vetri**, **Vittorio Ferrara**, and **Giuseppe Sancataldo**: Wrote the manuscript with contribution from all the authors. All authors approved the final version of the manuscript.

## Data Availability

Data available on request from the authors.
